# Current developments in forensic interpretation of mixed DNA samples (Review)

**DOI:** 10.3892/br.2014.232

**Published:** 2014-01-28

**Authors:** NA HU, BIN CONG, SHUJIN LI, CHUNLING MA, LIHONG FU, XIAOJING ZHANG

**Affiliations:** Hebei Key Laboratory of Forensic Medicine, Department of Forensic Medicine, Hebei Medical University, Shijiazhuang, Hebei 050017, P.R. China

**Keywords:** mixed DNA, short tandem repeat, mixture proportion, heterozygous balance, International Society of Forensic Genetics, statistical genetics

## Abstract

A number of recent improvements have provided contemporary forensic investigations with a variety of tools to improve the analysis of mixed DNA samples in criminal investigations, producing notable improvements in the analysis of complex trace samples in cases of sexual assult and homicide. Mixed DNA contains DNA from two or more contributors, compounding DNA analysis by combining DNA from one or more major contributors with small amounts of DNA from potentially numerous minor contributors. These samples are characterized by a high probability of drop-out or drop-in combined with elevated stutter, significantly increasing analysis complexity. At some loci, minor contributor alleles may be completely obscured due to amplification bias or over-amplification, creating the illusion of additional contributors. Thus, estimating the number of contributors and separating contributor genotypes at a given locus is significantly more difficult in mixed DNA samples, requiring the application of specialized protocols that have only recently been widely commercialized and standardized. Over the last decade, the accuracy and repeatability of mixed DNA analyses available to conventional forensic laboratories has greatly advanced in terms of laboratory technology, mathematical models and biostatistical software, generating more accurate, rapid and readily available data for legal proceedings and criminal cases.

## 1. Aim and scope

Twenty years after the first development of DNA fingerprinting, the forensic analysis of mixed DNA samples has only recently achieved the level of reliability required for individual identification in complex mixed DNA. Male-female mixed DNA samples in rape cases are commonly analyzed in forensic laboratories and, although less common, the need for effective analysis of male-male mixed DNA samples from multiple perpetrator (group rape) and homicide cases is increasingly prevalent. Although it is impossible to provide a completely exhaustive account of recent advancements in mixed DNA analysis, the aim of this review was to provide the reader with an understanding of the developments in this field over the last decade and the ongoing research required to improve the analysis of mixed DNA samples for forensic purposes.

## 2. Brief history

Forensic DNA analysis is a relatively young field, based on DNA-based identity testing first developed in 1985 (1). The first approaches to the application of DNA evidence to criminal cases involved calculating the percentage of the population excluded or included based on a DNA profile, which failed to consider the interactions between the number of alleles and contributors based on the numerical evidence provided in mixed DNA stains (2). As a result, these techniques commonly overstated the evidence against the defendant and were superseded in the mid-1990s by techniques using likelihood ratios (LRs) (2).

In a detailed historical account of the development of DNA-based identity testing, or DNA fingerprinting, Saad (3) reported that DNA profiling was implemented worldwide by 1986, following the introduction of amplification by polymerase chain reaction (PCR) and identification of numerous new 6- to 100-base repeating sequences referred to as minisatellites, or variable number tandem repeats (VNTR). Notably, one of the first forensic applications of these techniques was in the case of a double rape and homicide of two young girls in 1986, resulting in the clearing of one original suspect and the identification of the culprit using semen samples (4).

In the early 1990s, forensic DNA analysis moved from markers consisting of large core repeat units and overall large amplicon size (such as D1S80) to short tandem repeats (STRs) and the first widely available commercial kits for typing multiple STRs in a single reaction became available in the late 1990s/early 2000s (5). In contemporary forensic testing, VNTR and STR markers remain useful due to their polymorphic nature, wherein an individual expresses inherited markers from each parent in a Mendelian fashion (3). The analysis of complex DNA mixtures, particularly those containing several DNA profiles (>4), remains a challenge in modern forensic genetics, resulting in the recent development of laboratory techniques, mathematical models and software for improving these analyses (6).

### Modern guidelines for interpretation of mixed DNA samples

Significant improvements in sensitivity for trace samples in DNA mixtures have emerged over the last two decades (7). The theoretical issues involved in the accurate determination of individual profiles from mixed DNA samples have also been influential in policy and legal considerations. In order to standardize the optimal practices for examining DNA mixtures and low copy number (LCN) reporting, the DNA commission of the International Society of Forensic Genetics (ISFG) was convened in 2006, producing guidelines that include step-by-step analysis procedures still widely employed across the globe (8). Notably, responses to these guidelines that take into consideration national factors and produce more detailed guidelines for unacceptable practices have been proposed or published by the European DNA Profiling Group of the ISFG (UK), Technical DNA Working Group (UK) and ‘Stain Commission’ (Spurenkommission-gemeinsame Kommission der rechtsmedizinischen und kriminaltechnischen Institute; Germany) (7,8). Those papers further prompted the Biology Specialist Advisory Group (BSAG) of the Australian and New Zealand forensic science community to respond with publications aimed at improving overall laboratory quality, specifically focusing on the application of optimal techniques by research laboratories, even when not explicitly recommended by judicial requirements (9).

## 3. Laboratory analysis

### Sample types and characteristics

The quality of the samples is generally associated with the number of loci providing genetic information available for analysis in a given sample (10,11). Mixed DNA most commonly consists of an unknown DNA profile and another known DNA profile, most commonly from the victim. As the number of involved profiles increases, however, the discriminatory power may decrease (12). In practical forensic applications, the majority of mixed DNA samples consist of ≤4 different profiles (6). Forensic stain DNA profiles from crime scenes generally comprise 3 allele types: unmistakable alleles; alleles that may be masked by an artifact, such as a stutter; and alleles that have dropped out completely and are therefore not detectable (6).

In forensic examinations, it is crucial to identify DNA mixtures prior to any significant investment in analysis. The presence of >2 allelic bands per locus may be used to infer a mixture, although additional bands may also be present as a result of stutters or somatic/genetic polymorphisms (11). The second step in initial sample examination is the determination of allelic peaks, which should be within ±0.5 bp of the designated control allele ladder marker, and band shift, which should be approximately constant (12,13). These parameters are required for effective testing.

Forensic mixed DNA samples most commonly originate from semen and blood samples, although hair, skin, saliva, fingernails and buccal cells may also be tested. In rape cases, mixed male-female samples are the most common. These samples require more advanced analysis when complicated by the presence of multiple male contributors in the case of multiple perpetrators (group rape) and increasingly prevent crime garnering legal attention worldwide (14). In several cases, such as rape, significantly larger amounts of victim DNA are present. In addition, the degradation of suspect DNA due to a prolonged time period between the occurrence of the crime and DNA sampling may also reduce the quality of one DNA component in a mixture (5). Thus, forensic samples may comprise a complex mixture of numerous unique and overlapping major and minor components from different individuals that must be identified for accurate analysis.

### Identifying the number of DNA profile contributions

With the currently available STR technology applying known polymorphisms, it is impossible to attain the number of contributors in a DNA sample with 100% certainty, due to possible DNA masking effects (11). Generally, the presence of multiple contributors is identified by the maximum allele count strategy, a relatively simple strategy that is easy to present in court (15). However, when ≤2 alleles are observed at any locus in a profile, a sample may still represent a DNA mixture (6).

Biedermann *et al* (11) proposed two probabilistic approaches for solving these issues using a deterministic analysis of the minimum number of contributors to ‘explain’ the allele set or, alternatively, Bayes’ theorem applying a probability distribution for a set number of contributors. Haned *et al* (15) proposed a predictive value (PV) that may be applied as a global measure of likelihood-based estimator efficiency, useful in measuring the uncertainty associated with estimates in mixed DNA samples. Although these techniques are more complex, which has made certain researchers hesitant to present these findings in court, modern methods for the determination of contributor number should not be overlooked by forensic experts, who should be aware of alternatives to the conventional maximum allele count strategy (15).

In several cases, the number of participants is known; however, particularly in cases of rape, the presence of multiple DNA profiles must be carefully considered to avoid error. In particular, rape cases involving victim and suspect DNA may be complicated by the presence of low levels of DNA from a consensual partner, which may be overlooked in up to 3% of such cases using modern techniques (10). Buckleton *et al* (10) described a method for measuring quantitative effects in order to reveal if an apparent mixture of n individuals is actually an n+1 person mixture.

### Special considerations for mixed DNA samples: stutter, LCN, drop-out and contamination

Stutter bands are characterized by the presence of one tandem repeat less than the parent allele and these occurrences may compound mixed DNA analysis (11). Additionally, in samples with small traces, LCN (<200 pg) PCR may result in the occurrence of stochastic effects that produce allelic imbalance and drop-out, invalidating the conventional rules for analysis of heterozygous balance and other DNA characteristics (6). Notably, the most common cause of LCN is the use of high numbers of PCR cycles (>28 cycles) in major/minor component mixtures. Therefore, LCN mixture analysis should allow for stochastic events, including drop-out, heterozygous imbalance and contamination (16). The ISFG recommended that, in relation to LCN, stochastic effects may limit the usefulness of heterozygous balance and mixture proportion estimates, particularly in consideration of allelic drop-out and drop-in (contamination) (6).

DNA may also be introduced after it is collected from the crime scene (drop-in), resulting in the presence of unrelated DNA from investigating officers, laboratory technicians and laboratory plasticware (17,18). These contaminations are often difficult to identify, making determination of mixed DNA in forensic samples prior to testing critical to ensuring accurate results.

### Improvements in forensic laboratory techniques

In order to apply DNA testing to more criminal cases, attaining higher discriminatory power between individuals is required. To accommodate this need, the number of loci targeted by single multiplex systems has increased over the last decade, producing a number of commercially available and well-validated kits for forensic use that incorporate 15–16 highly variable STR loci (plus amelogenin), including the PowerPlex^®^, ESX and ESI systems, as well as AmpFlSTR^®^ NGM (5). Notably, these recently commercialized kits are well-known for their use of improved primer designs, buffer compositions and amplification conditions, all aimed at allowing maximum determination from trace samples (5).

Recent laboratory techniques have also been designed with the goal of improving quantification and limiting the use of samples in quantification determination. The quantification of total human and male DNA in complex forensic samples has been achieved using the Plexor^®^ HY system, providing critical information on how to proceed with sample analysis and whether interpretable STR results may be expected (19). In quantifying DNA in samples, the goal of the user has been suggested to be the primary determinant for method selection, as different approaches provide either improved accuracy (single-copy approaches) or better sensitivity (multiple-copy approaches), as demonstrated in a comparison of autosomal and Y chromosomal DNA using Quantifiler^®^ Duo with TaqMan^®^ versus Plex or HY1 with primer quenching assay and multi-copy probes (20).

In rape cases, modern techniques allow for the amplification of small amounts of suspect DNA up to 24 h after the crime, using the AmpFlSTR^®^ SGMPlus^®^ multiplex kits, even under LCN conditions where the female (victim) DNA is present in a significantly higher proportion (14). Unfortunately, several forensic facilities face enormous backlogs of samples from sexual assault cases. However, modern techniques using rapid alkaline lysis for DNA extraction from sexual assault evidence have been reported; these techniques may generate purified sperm fraction extracts yielding STR typing. The results of these methods were shown to closely approximate those obtained from conventional organic/dithiothreitol differential extraction (21). The wide application of such procedures may increase the availability, reduce the cost and decrease the turnaround time for forensic testing in cases of sexual assault.

In cases of violent assault, forensic DNA samples collected from under the fingernails may be tested, most commonly through the use of Qiagen™ extraction and AmpFlSTR^®^ SGM Plus amplification. Such profiles produced appreciable amounts of foreign DNA in only 13% of the samples after 24 h in a previous report (22), although significantly higher levels of DNA may be found in cases of prolonged exposure to the foreign DNA (such as in the case of foreign DNA from cohabitating roommates, partners, or children living with the subject) (23,24). In methods similar to those applied in the identification of foreign DNA beneath the fingernails, suspect DNA was effectively identified from mixed DNA samples taken from the saliva up to 60 min after intense or constrained kissing, using a high-sensitivity DYS14 assay (25). A number of novel techniques were also recently described for aneuploidy detection, useful in forensic identification (26).

### Amplification of trace DNA from mixed samples

The most striking advancement in forensic DNA analysis over the last decade is the ability to identify individuals using STR profiles produced from very small sample amounts (trace samples) (5). One strategy for achieving higher sensitivity for trace samples is improving the amplification methods. Notably, cell lysis is required prior to DNA extraction, which may also present a source of DNA loss due to the interference of cellular materials (27).

For relatively pristine forensic samples, laser microdissection and fluorescence *in situ* hybridization have been applied as more sensitive methods for producing DNA materials with minimal interference from cellular material for STR analysis, successfully employed by the Forensic Science Service in sexual assault cases (27,28). When subjected to a one-tube extraction and amplification method, full Identifiler^®^ profiles for semen and epithelial cells were produced using this method (28). Particularly in the case of male-male mixed DNA, haplotype-specific extraction with optimized buffers has been proposed as a more straightforward method for DNA mixture analysis, producing improved enrichment of male DNA from single contributors (29).

Alternatively, high-density single-nucleotide polymorphism (SNP) genotyping arrays may achieve greater sensitivity. In a previous study, upwards of 500,000 markers were used to exploit raw allele intensity measurements, achieving reliable detection in trace fractions of <0.1% from mixtures with a large number of contributors (30). Similarly, Voskoboinik and Darvasi (31) leveraged the value of unique rare allele combinations using a specially designed panel of 1,000–3,000 SNPs, each with a relatively low (<0.1) minor allele frequency. This method was shown to be effective in identifying individual DNA profiles in complex mixtures of ≤10 individuals.

## 4. Software systems

### Software development

Modern technologies, including dynamic and web-based software designs, have only recently began to be applied for practical forensic use, producing more accurate analysis of complex samples comprising partial DNA profiles. These samples often include missing alleles (allelic drop-out) or additional alleles (drop-in), which remains a challenge. In order to solve these problems in mixed DNA analysis, MasterMIX, a freeware solution to aid in the interpretation of mixtures using peak height/area information, and DNAMIX software systems (http://www.genomine.org/dnamix/index.html) were developed in 1998–1999 for the qualitative analysis of mixed DNA samples using comparative reference methods (32). The comparative reference method involves the qualitative assessment of mixed DNA profiles obtained directly by simple visual inspection, so as to exclude suspects. Considering that this method does not quantitatively assess biological materials, identification conclusions do not meet the requirements for forensic evidence. In 2005, MIX05 (http://www.cstl.nist.gov/strbase/interlab/MIX05/MIX05poster.pdf) was developed by the US National Institute of Standards and Technology for quantitative analysis of mixed DNA samples from only two individuals. MIX05, however, is limited by its inability to exclude influencing factors during analysis, such as stutter bands, drop-outs, drop-ins and low-copy DNA. As a result, the program DNA_Data Analysis was subsequently proposed (33), which can only calculate the mixture proportion (M_x_) of DNA components in mixed DNA samples from three individuals.

In 2007, LoComatioN software was developed to target low-copy DNA profiling in mixed DNA analysis (34). This software established a consensus method and simultaneously estimated the population substructure, probability of allelic loss and probability of allelic contamination drop-in; it did not, however, make use of the peak height/peak area in mixed DNA profiling for quantitative analysis. Shortly thereafter, the commercial solution GenoProof^®^ Mixture was released as an all-in-one solution for forensic DNA sample analysis allowing for high throughput, reducing the need for additional software. In 2009, the GeneMarker^®^ HID software was released to aid in the identification of complex mixture using the widely accepted guidelines set forth by He *et al*, thus reducing unnecessary repetition in high-throughput laboratories (35).

The GeneMapper^®^ ID-X Mixture Analysis (GeneMapper Corporation) software tool was released in 2011. This program was developed to conduct complex quantitative analysis of mixed DNA samples from two individuals using two quantitative assessment parameters: M_x_ and heterozygous balance (H_b_) (36). This tool calculated the intensity of mixed DNA evidence using the LR method recommended by the ISFG. However, uncertain factors such as stutter bands, allelic loss, shared alleles, low-copy DNA and DNA contamination may still affect the reliability of the results from mixed DNA analysis using this tool.

In the same year, the TrueAllele^®^ software was developed, establishing a probability profiling method using a mathematical model containing validation and quantitative probability modeling methods (37). This software is almost able to perform quantitative analysis of mixed DNA samples from three individuals; however, increased uncertainty in the analysis of mixed DNA samples with low signals remains a problem. In addition, there is no appropriate threshold for an inclusion log (LR) when suspects cannot be excluded as the source of mixed DNA sample. Thus, the analysis of >4 individuals remains challenging.

Open-source initiatives recently produced Forensim, an R statistical software that interprets forensic DNA evidence and reports the weight of that evidence. In fact, Forensim is the first such open source interpretation tool, available freely from http://forensim.r-forge.r-project.org (38). Recently, LRmix software applying a Forensim package was applied to analyze complex DNA mixtures (39).

### Comparative DNA databases

DNA databases are a critical tool for solving crimes based on DNA evidence when other evidence, such as fingerprints, is unavailable or inconclusive. Using DNA evidence, investigators may identify suspects by searching for a DNA perfect match; however, large and comprehensive networked databases of DNA profiles were required to realize the benefits of DNA profiling. The first national DNA database became operative in the Unites States in 1995 and by June, 2011, the US Federal DNA database had been used in >141,300 investigations to produce >147,200 hits (12). Although the use of a fixed number of contributors as an output allows for more useful results generated by these databases, it has been suggested that these ‘easy’ techniques may constrain the usefulness of comparative DNA databases as improved laboratory and biostatistical methods become available (17).

The DNA Identification Act of 1994 authorized the US Federal Bureau of Investigation (FBI) to expand its pilot DNA identification project into a national DNA database, the Combined DNA Index System (CODIS), combining DNA analysis with computer technology (40). This database consists of a Forensic Index, containing crime scene DNA profiles, and an Offender Index, containing the profiles of convicted criminals (40). Currently, the FBI applies a standard set of 13 specific STR regions in its CODIS database, which stores local, state and national DNA profiles from convicted offenders, unsolved crime scene evidence and missing persons (41). In this database, the odds that more than one individual share a 13-loci DNA profile is approximately one in one billion (41). Similarly, Canada established a CODIS-based National DNA Databank in 2000, composed of the Convicted Offender Index and Crime Scene Index (42).

In criminal investigations, multiple DNA samples may be compared against the DNA profiles included in these databases to produce ‘cold hits’ in order to identify suspects (43). However, complex mixtures often produce numerous results from database searches, necessitating high time and resource involvement, previously limiting the usefulness of these resources in high-profile cases. Recently, however, novel strategies were employed to reduce the number of punitive database hits by using mixture interpretation and review of original electropherograms, thus minimizing the risk of adventitious hits and making database searching more practical for application in criminal investigations (42).

### Database-based identification of individuals by partial matching and familial searching

Ideally, searching a DNA database will produce a perfect match for an individual, allowing for a definitive identification based on DNA evidence (12). However, when no perfect match is found, investigators often search for approximate matches that may indicate family relationships by evaluating the LR of relationships between individuals, shown to produce effective results in ~70% of investigations (12). Generally, a partial match is considered when profiles share at least 15 STR alleles with an offender, although the policies for partial matching vary by jurisdiction (44).

Evett and Weir provided a formula that is used to examine the LR for two-person relationships (44), producing a satisfactory discriminatory power for examining DNA mixtures when at least one DNA profile (victim profile) is known (12). However, although the application of familial searching may produce useful DNA results, issues of policy may compound the use of such resources. For example, California’s familial search policy limits testing to ≤168 candidates using Y-STR typing (45).

### Controversial use of databases

Notably, there is an ongoing debate as to the effectiveness of comparing database entries between jurisdictions and through time. As several new countries have established national DNA databases, the forensic community was required to select a core set of loci for DNA profiling (46). A small set of core loci mainly consisting of tetranucleotide tandem repeat loci was proposed based on those commonly used by most jurisdictions, producing the largest possible overlap (47,48). Although this consensus allows for easier comparisons of profiles, some suggest that it may stifle the use of innovative new technologies for quality and efficiency improvements (48), such as moving from STRs to SNPs more adaptable to high-throughput and miniaturized typing systems. Whilst sensitive SNP-based individualization profiling systems are currrently available, modern databases may prohibit them from routinely being employed (5,48). Additionally, the ease of applying software that provides a fixed number of contributors as an output is appealing, allowing for calculation of allelic probability in a single step (30). However, this benefit may not be sufficient to justify employing these techniques, due to the high number of false determinations and large resource involvement required for identifications useful in court (30).

### Automated extraction scripts

The need for development of computer-based expert systems that may assist in the interpretation of complicated DNA profiles has recently been recognized (7). Automation may also be helpful in handling the large number of hits possible when conducting database searches using multiple DNA mixtures. One such method has been proposed that involves automated generation of DNA profiles from replicate PCRs by combining composite and consensus methods using a bracketed system, wherein the allelic balance threshold is used as a variable to separate DNA profiles of major and minor donors (49). The automated extraction of dominant profiles has the ability to save considerable time in producing composite-consensus profiles and drop-in/drop-out rates may be easily compared. There is currently a need for bioinformaticians and statisticians to develop open-source scripts that may be widely employed in forensics laboratories (49).

## 5. Mathematical modeling

### Probability approach and LR

The accurate biostatistical interpretation of mixed DNA profiles remains a challenge to contemporary forensic researchers, particularly in cases where DNA profiles are incomplete (7). The two most common approaches employed to analyze mixed DNA samples are i) the classical profile probability approach and ii) the LR approach (6). The use of LRs to identify familial associations has been well described in several reviews (50–52). In the context of forensics, the profile probability approach is used to generate an evidentiary DNA profile (43) following a stated hypothesis (H_o_) (i.e., person is or is not a suspect), generally written as: Pr (E|H_o_); where Pr is ‘probability’ and the vertical line (conditioning bar) is ‘given’.

When two or more hypotheses are suggested, the LR may be generated as follows:

$$LR=\frac{Pr(E|H_{p})}{Pr(E|H_{d})}$$

where the prosecution hypothesis (H_p_) and the defense hypothesis (H_d_) are represented. An LR>1 favors the prosecution and, accordingly, an LR<1 favors the defense (6). The ISFG has provided detailed guidelines regarding the application of the LR method using the unrestricted combinatorial approach (not taking account of peak height/areas) and the restricted combinatorial approach (taking account of peak height/areas) for the analysis of mixed DNA samples (6). Notably, the probability of exclusion, or random man not excluded (RMNE), as well as the complementary probability of inclusion, propose a binary view of alleles as either present or absent. This presents the implicit assumption that masking is not occurring (6).

According to BSAG, LR is the preferred approach for the interpretation of mixed DNA samples, whereas the RMNE approach should be restricted to unambiguous DNA profiles (9). However, novel approaches have been suggested to improve the robustness and reliability of LR testing in complex mixtures. For example, a case-specific ‘Tippett’ test was proposed to provide additional robustness parameters for LR identity determination, which, although complex, may better account for drop-out and contamination (53). To further improve these methods, it was suggested that probabilistic weighting should be applied to each possible genotype rather than simply applying a zero/one weighting, as is inherent in the restricted combinatorial (binary) approach (8).

### Maximum allele count and Bayesian methods

Bayesian networks were recently employed for the study, development and implementation of probabilistic procedures for evaluating the probative value of scientific evidence in forensic cases (53). The widely used maximum allele count method owes a significant proportion of its popularity to its ease and rapidity of application, although it is generally most effective for a low number of contributors (11). Biedermann *et al* (11), however, proposed the use of Bayesian inference over maximum allele count methods due to its ability to produce fewer false determinations when multiple contributors are present, although its complexity is notably higher.

Bayesian networks are a pictorial representation (dependencies and influences = arcs; variable relationships = nodes) of data from genetic markers using Mendelian laws and logical associations between genes and genotypes constructed by applying graph and probability theory to a probabilistic inference problem (54). These techniques are widely applicable to criminal identification, relatedness testing, database searching and trace evidence evaluation, such as that required for the analysis of mixed DNA or small-quantity DNA stains, detailed in the review by Biedermann and Taroni (54). Object-oriented Bayesian networks have been widely applied in forensic analysis (55–58). Notably, joint Bayesian analyses using gamma distribution were also successfully applied for identifying trace DNA samples in multiple mixed DNA samples (59).

## 6. Conclusions and recommendations for forensic mixed DNA testing

Over the last decade, significant advancements were made in laboratory practices, software systems and mathematical models for the practical analysis of mixed DNA samples. The methods for the analysis of mixed DNA samples often yield low detection rates, reducing their practical usefulness in criminal investigations due to failure to meet the relevant legal standards provided by court systems. However, improvements in amplification and sensitivity of testing have facilitated the use of DNA testing in more criminal cases, such as rape, where only very small amounts of suspect DNA may be mixed with victim DNA. These techniques may also significantly enhance the ability of forensic investigations to product legally useful evidence when presented with mixed DNA samples from the saliva, vaginal swabs and under the fingernails that comprise DNA from several minor contributors, such as in cases of group rape.

Despite the significant advancements achieved, ongoing enhancements in combinatorial methods are required to account for all the challenges, including those presented by stutter, contamination and artifacts of allelic drop-out, in accordance with ISFG recommendations (6). Therefore, more sophisticated probabilistic approaches are required, particularly for samples containing DNA from a number of different individuals. As these methods become increasingly complex, the need for automation and software becomes more urgent, as evidenced by the recent proliferation of the biostatistical industry in this field. In the near future, such forensic scripts may be expected to appear among open-source programming and are likely to become standard laboratory tools.

Particularly in male-male DNA samples provided in rape cases, the measurement and statistical analysis of fluctuation and stability of each index is critical for identifying a specific genotype. Modern experimental models minimize error and maximum stability in the H_b_, M_x_ and M_r_ parameters for the average peak height of active alleles, providing a basis for improved analysis. Notably, high-quality autosomal STR profile analysis using multiplex PCR systems produces peak balance among alleles. In practice, variations in signal intensity may occur between heterozygous alleles as a result of uneven amplification or peak unbalance. In forensic casework, significant perturbations in peak height/area balance are most often attributed to the presence of shared alleles, indicating a complex mixed DNA stain, rather than preferential amplification of alleles during PCR. Although some variations in balance in heterozygotes are to be expected, accurate individual identification in complex mixed DNA profiles requires that STR loci be selected more rigorously to avoid inaccuracy. Notably, not all STR markers suitable for single DNA samples are appropriate for the analysis of mixed DNA samples, particularly in cases where degradation of some or all the profiles may be advanced.

As the ISFG suggested, there is also a need to further standardize working practices for the interpretation of DNA profiles for accreditation according to recognized laboratory standards, such as ISO 17025 (7). Furthermore, forensic laboratories may require ongoing training to stay relevant. Each laboratory should individually assess their processes in order to ensure that the results are optimal, particularly in cases of complex DNA mixtures. Notably, common laboratory practice often entails bounding contributor numbers to the minimum required to explain the observed DNA profiles. To achieve improved results, the laboratories should make use of all data, rather than just alleles per locus, in order to ensure that a sample is not a more complex mixture than initially assumed (60). A recent study investigated several methods to improve the identification of multiple DNA mixtures, including a promising alternative approach based on the maximum likelihood principle (61). Further development and implementation of these methods into routine forensic laboratories may be necessary to achieve improved accessibility to accurate mixed DNA identification processes.

The modern use of Bayesian networks is a first step toward a more cost-effective and sensitive detection of individual profiles in multiple DNA samples. However, the application of Bayesian networks remain limited by several basic assumptions, including that of independence within and across markers and that all unrepresented individuals stem from a homologous population (52). Future investigations are required to create models with fewer assumptions that are more widely applicable. These improved Bayesian models should also be designed in consideration of artifacts, such as stutter and allelic drop-out, that often represent a problem in mixed DNA samples (54).

Additionally, in the experience of the authors, the practical applications of common ABI ID kits, a widely accepted genetic marker system for individual identification, are not always effective for individual identification for forensic or paternity purposes. Thus, we suggest that not all samples containing genetic markers are suitable for mixed DNA analysis using the currently available methods. Instead, a genetic marker system suitable for mixed DNA analysis is required, as is the development of customized kits designed for individual identification and paternity testing using mixed, trace and epigenetic DNA samples.

The currently available methods for mixed DNA samples remain limited due to the failure of the international standards for DNA testing to recognize the developing field of software for mixed DNA analysis. Since multiple factors are involved in the development and application of these software systems, the current software and automation systems, as well as the evolution of experimental technology, should be considered in future research. Third-generation sequencing technology has also developed significantly, utilizing a combination of sequencing technologies with a variety of genetic markers to identify individuals in mixed DNA samples (29), although these techniques require further investigation. In the near future, researchers and decision-makers should give careful consideration to the application of such software systems in practical laboratory settings.

In summary, the role of mixed DNA samples as forensic evidence has become a focus of investigation, challenging forensic researchers to produce more rapid, reliable and easy-to-understand methods for legal applications. Notably, accurate profiling of mixed DNA samples in group rape cases resulting in several mixed DNA profiles is urgently needed. As these samples are increasingly relevant in forensic examinations related to criminal cases, it is of great significance to develop innovative experimental techniques and software appropriate for complex mixed DNA analysis.
